# Effects of biochar on the fate and toxicity of herbicide fenoxaprop-ethyl in soil

**DOI:** 10.1098/rsos.171875

**Published:** 2018-05-02

**Authors:** Xu Jing, Tengfei Wang, Jiali Yang, Yanli Wang, Huifang Xu

**Affiliations:** College of Food Science and Engineering, Shanxi Agricultural University, Taigu, Shanxi 030801, People's Republic of China

**Keywords:** fenoxaprop-ethyl, degradation, toxicity, biochar, earthworms

## Abstract

Biochar, as a soil amendment in agriculture, has attracted considerable attention. In the study, the fate and toxicity of the herbicide fenoxaprop-ethyl were evaluated in soils with and without 5% rice husk biochar amendment. Fenoxaprop-ethyl and metabolite fenoxaprop degradation followed first-order kinetics in the two soils. Fenoxaprop-ethyl decreased fast with half-lives less than 2 days. Large amounts of fenoxaprop formed and remained in the control soil. However, fenoxaprop was much lower in the biochar-amended soil with reduction over 85% on the 35th day. The estimated half-lives of fenoxaprop were 56.9 and 1.5 days in the control and biochar-amended soils, respectively. Biochar restrained the formation and promoted the dissipation of fenoxaprop. Biological indicator earthworms (*Eisenia fetida*) were used in a 14-day acute toxicity test. Fenoxaprop-ethyl showed low toxicity to earthworms with LC_50_ value of 322.9 µg g^−1^. Biochar amendment was non-toxic to earthworms and effectively reduced the toxicity. The results suggested that the application of biochar may reduce the risks of fenoxaprop-ethyl in the soil environment.

## 1. Introduction

**Figure 1.**
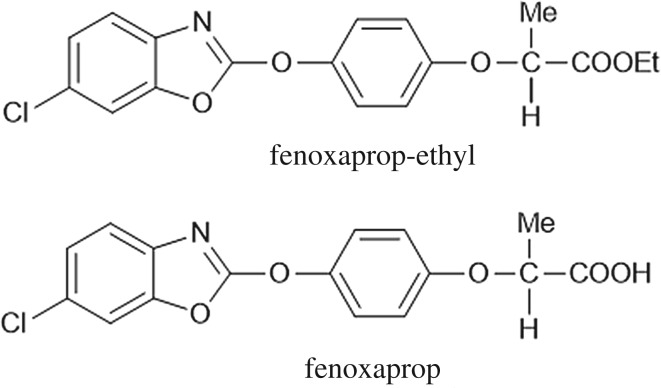
Chemical structures of fenoxaprop-ethyl and fenoxaprop.

Fenoxaprop-ethyl is one of the most widely used herbicides to control soybean and wheat weeds in many countries [1]. Fenoxaprop-ethyl (figure 1), ethyl 2-[4-[(6-chloro-1,3-benzoxazol-2-yl)oxy]phenoxy]propanoate, inhibits acetyl coenzyme A carboxylase (ACCase), thereby inhibiting the synthesis of fatty acids and the growth of weeds and eventually leading to death [2]. Most pesticides applied to farmland are converted into one or more metabolites via physical, chemical and biological processes. Actually, the metabolites frequently accumulate to a greater extent in organisms and exhibit greater toxicity than parent compounds [3]. Fenoxaprop-ethyl may hydrolyse to its related acid fenoxaprop (2-[4-[(6-chloro-1,3-benzoxazol-2-yl)oxy]phenoxy]propanoic acid, figure 1). The metabolite fenoxaprop also has herbicidal activity and shows stronger activity in inhibiting ACCase in weeds [4]. Fenoxaprop-ethyl and fenoxaprop residues adversely affect soil ecosystems and bioaccumulate in crops, causing potential harms to human health through the food chain [5].

The fate of fenoxaprop-ethyl in various soils has attracted much attention. It was reported that fenoxaprop-ethyl dissipated in soil with half-lives of 0.5 day [6]. The soil residues of fenoxaprop-ethyl were below detectability within 6 days in a field study [7]. Smith [8] reported that fenoxaprop-ethyl was almost converted to fenoxaprop within 24 h in soils with high water content under laboratory and field conditions. Low water content could slow down the degradation of fenoxaprop-ethyl. Zablotowicz *et al.* [9] studied the role of soil pH on degradation of fenoxaprop-ethyl. Twenty-five per cent and 55% of fenoxaprop-ethyl hydrolysed to fenoxaprop within 20 h in acidic and neutral soils, respectively. Chen *et al*. [10] studied the degradation of fenoxaprop-ethyl in soils from two geographically distinct regions, whose half-lives were 1.8 and 2.4 days.

Biochar research has progressed considerably in recent years [11]. Biochar is a solid material thermochemically converted from biomass under limited oxygen conditions. Biochar can be processed from a wide range of sources and has porous carbonaceous structures with functional groups. Biochar as a soil amendment can improve soil fertility, restore degraded land, reduce carbon emissions and increase crop yields [11]. Biochar not only has the potential to change physical and chemical properties of soil but also the soil microbial activity, community and enzyme activity [12]. Pesticides are widely used on crops and most remain in soil, posing potential risks to the soil environment [13]. Biochar may significantly impact the environmental behaviours of pesticides in soil. On the one hand, due to the porous structure and large surface area, biochar sorption may decrease the bioavailability of pesticides to soil microorganisms and slow down the biodegradation [14]. On the other hand, biochar may increase microbial activity by providing available carbon source and other nutrients (N, P and K), and ultimately accelerate the biodegradation of pesticides in soil [15]. Therefore, impacts of biochar on the fate of pesticides in soil and the mechanism still need to be further studied. There has been intensive research of biochar in the field of remediation and sorption [16], but little literature involved in the environmental behaviours of metabolites.

In addition, less attention has been paid to the effects of biochar on toxicity of pesticides to soil biota [17]. Earthworms, an essential part of the soil fauna, make direct and indirect contributions to soil ecosystems, such as soil structure, fertility, microbial biomass and enzymatic activity [18]. Earthworms serve as a major food source for many animals, such as birds, reptiles and mammals. It has been used as an important bioindicator organism in soil ecotoxicological research studies [19]. Fourteen-day acute toxicity bioassays were the most frequent studies, aimed at assessing the potential risks of environmental contaminants in soil [20]. Effects of biochar on soil properties may bring negative and positive effects on earthworms. There has been research on the effects of biochar addition on the survival of earthworms [19,21,22]. However, the study of combined toxicity effects of biochar application and pesticides to earthworms has been very limited.

To guide the rational application of biochar, the potential risks of returning biochar to soil need to be identified. In this study, the effects of biochar amendment on fenoxaprop-ethyl residues in soil were evaluated. The content of metabolite and the toxicity of fenoxaprop-ethyl to soil organism earthworms were fully considered. Assessing the impacts of biochar on pesticide risk is essential for protecting the environment and human health.

## 2. Material and methods

### 2.1. Chemicals and materials

Fenoxaprop-ethyl with purity 98.0% was obtained from Institute for the Control of Agrochemicals, Ministry of Agriculture (Beijing, China). Fenoxaprop with purity 98.0% was achieved by synthesis according to the literature [23]. Chromatography-grade acetonitrile was bought from Fisher Scientific (Fair Lawn, NJ, USA). Water was purified by a Milli-Q system (Bedford, USA). Analytical-grade acetone and ethyl acetate were purchased from Beijing Chemical Works (Beijing, China).

The biochar was produced from rice husks and subject to pyrolysis at 550°C. The biochar contained 52.2% carbon, 1.6% hydrogen, 1.6% potassium, 0.8% phosphorus, 0.7% nitrogen and 0.2% sulfur.

The soil was collected from Baiwang Mountain Forest Park (Beijing, China). The soil was air-dried at room temperature and passed through a 2 mm sieve. The soil contained 6.9% clay, 40.8% silt and 52.3% sand. The organic matter was 4.0% and pH 7.1.

Earthworms (*Eisenia fetida*) weighting 0.4 ± 0.05 g were obtained from a farm, Beijing, China. Earthworms were acclimatized to laboratory conditions for 14 days with 20 ± 2°C under a 12 L : 12 D cycle.

### 2.2. Degradation experiments

The moisture content of the soil and biochar was adjusted to 30%. In the control treatment, 500 g of the soil was weighed. In the biochar-amended treatment, the dose of biochar was set at 5%, corresponding to 25 g of biochar and 475 g of the soil weighed. The applied dose of biochar corresponds to the doses administered in other studies conducted with biochar [17]. The concentrations of fenoxaprop-ethyl in both treatments were set at 10 µg g^−1^ by mixing 1 ml of 5000 µg ml^−1^ of fenoxaprop-ethyl acetone solution. The following fortification method was adopted. Ten grams of the control soil or biochar-amended soil was first transferred into a beaker and stirred thoroughly. Then 1 ml of fenoxaprop-ethyl acetone solution was added. Another 490 g of control soil or biochar-amended soil was added gradually and stirred thoroughly. The glass beakers were placed in an incubator with 12 L : 12 D regimen at 20 ± 2°C. Five grams of the soil was collected at 1, 3, 5, 12 h, 1, 2, 3, 5, 7, 10, 14, 17, 21, 28, 35 days and stored at −20°C. There were three beakers for both control treatment and biochar-amended treatment at each time point. The soil in each beaker was collected only once.

### 2.3. Sample extraction and analysis

One gram of soil samples was placed in a 15 ml polypropylene centrifuge tube containing 4 ml of ethyl acetate. The soil was vortexed for 10 min and then centrifuged at 3500 r.p.m. for 3 min. The extraction was repeated again with 4 ml of ethyl acetate. The supernatant was collected and evaporated to dry under a nitrogen stream. The extracts were reconstituted with 1 ml of acetonitrile, passed through a filter, and analysed by HPLC-MS/MS.

HPLC-MS/MS analysis of fenoxaprop-ethyl and fenoxaprop was performed using Thermo Scientific TSQ Quantum Access Max equipped with an Agilent Eclipse XDB-C18 column (5 µm, 4.6 × 150 mm). The column temperature was maintained at 25°C. Using an autosampler, 5 µl aliquots were injected. The mobile phase consisted of acetonitrile and water (9 and 1 by volume) at a flow rate of 0.5 ml min^−1^. The mass spectrometer detector was operated in ESI mode. Spray voltage was 3500 V. Ion sweep gas pressure, sheath gas pressure and auxiliary gas pressure were 0, 35 and 15 arb. units, respectively. Vaporizer temperature and capillary temperature were 200 and 350°C, respectively. Retention times, ion modes, fragmentations, collision energies of fenoxaprop-ethyl and fenoxaprop are shown in table 1. The average recoveries for fenoxaprop-ethyl and fenoxaprop at three levels (0.1, 1 and 10 µg g^−1^) were between 85% and 102% in the control soil, and between 83% and 106% in the biochar-amended soil based on RSD below 20% (*n* = 3).

**Table 1.** HPLC-MS/MS parameters of fenoxaprop-ethyl and fenoxaprop.

compound	retention time	ion mode	parent (*m/z*)	product (*m/z*)	collision energy
fenoxaprop-ethyl	5.0	positive	362.1	288.1 244.0	17 23
fenoxaprop	3.6	negative	332.0	260.0 152.0	36 25

A one-phase exponential decay equation (*C_t_* = *A*_1* *_*+ A*_2_e^−*kt*^) was used to fitting the degradation of fenoxaprop-ethyl and fenoxaprop in soils [24]. Where *t* is the time (days); C*_t_* is the concentration in soil at time *t* (μg g^−1^); *k* is the degradation rate constant (day^−1^); *A*_1_ and *A*_2_ are constants (μg g^−1^). The statistical difference between control treatment and biochar-amended treatment was assayed using analysis of variance (ANOVA). The differences were considered statistically significant when *p* was less than 0.05.

### 2.4. Acute toxicity experiments

To evaluate the effects of biochar on the toxicity of fenoxaprop-ethyl, an acute toxicity test was conducted using the earthworms. According to the OECD guideline for testing of chemicals 207, earthworms were exposed to the fenoxaprop-ethyl contaminated soil for 14 days. The maximum exposure concentration was set to 1000 µg g^−1^ as recommended. Based on the pretests, the geometric exposure concentrations of fenoxaprop-ethyl in soil were set to 62.5, 125, 250, 500 and 1000 µg g^−1^. Each exposure treatment consisted of four replicates, each consisting of 10 earthworms and 750 g of the contaminated soil (30% moisture content) in a 1 l glass beaker. Solvent control groups were also conducted. The glass beakers were placed in a lighted incubator with continuous illumination at 20 ± 2°C. On the 14th day of exposure, mortality was recorded. The judgement of death was based on the response of the front end to mechanical stimulation. The acute toxicity bioassays were conducted in soil with and without biochar amendment. Biochar was amended and replaced 5% of the soil in glass beakers to study the impacts of biochar on the toxicity of fenoxaprop-ethyl on earthworms.

## 3. Results and discussion

### 3.1. Fate of fenoxaprop-ethyl in the control soil

**Figure 2.**
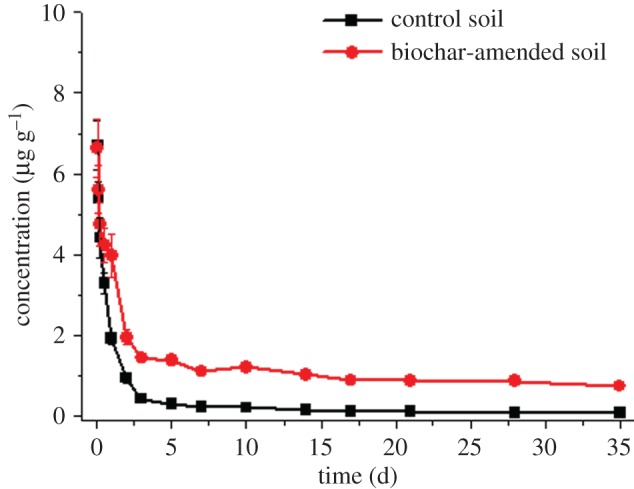
Fate of fenoxaprop-ethyl in control and biochar-amended soils.

Figure 2 illustrates the fate of fenoxaprop-ethyl in the control soil. Fenoxaprop-ethyl degradation followed first-order kinetic equation *C_t_ =* 0.215 + 6.370 × 10^−1.431*t*^ with *R*^2^ of 0.986 (table 2). The half-life of fenoxaprop-ethyl in the control soil was 0.5 day, which was consistent with the data reported in literatures [6,9]. After 2 days, the reductions of fenoxaprop-ethyl exceeded 90%. On the 35th day, the concentration of fenoxaprop-ethyl was 0.071 µg g^−1^ indicating a decrease of approximately 99%. There was low residual risk of fenoxaprop-ethyl in the control soil.

**Table 2.** First-order kinetic equation, half-lives (*T*_1/2_) and coefficient (*R*^2^) of fenoxaprop-ethyl and fenoxaprop in control and biochar-amended soils.

compound	soil	first-order kinetic equation	*T*_1/2_ (day)	*R*^2^
fenoxaprop-ethyl	control	*C_t_ =* 0.215 + 6.370 × 10^−1.431*t*^	0.5	0.986
	biochar-amended	*C_t_ =* 0.982 + 5.177 × 10^−0.75*8t*^	1.2	0.968
fenoxaprop	control	*C_t_ =* 2.354 + 3.269 × 10^−0.035*t*^	56.9	0.933
	biochar-amended	*C_t_ =* 0.686 + 2.965 × 10^−0.620*t*^	1.5	0.953

**Figure 3.**
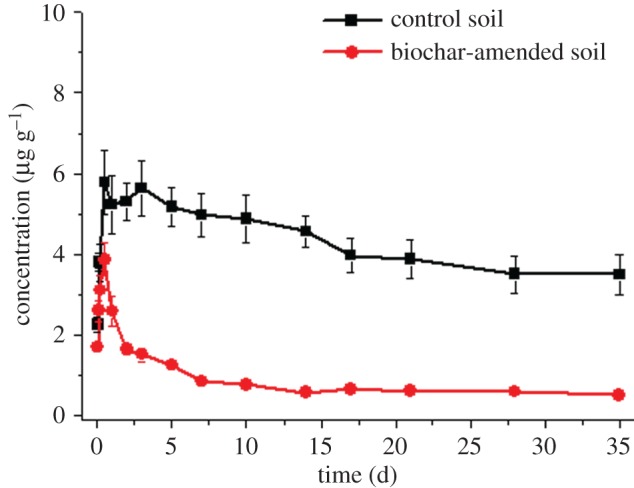
Fate of fenoxaprop in control and biochar-amended soils.

Large amounts of metabolite fenoxaprop were formed with the degradation of fenoxaprop-ethyl (figure 3). Fenoxaprop first increased rapidly to the maximum concentration of 5.787 µg g^−1^ at the 12th hour. Then fenoxaprop decreased slowly following first-order kinetic equation *C_t_ =* 2.354 + 3.269 × 10^−0.035*t*^ with *R*^2^ of 0.933 (table 2). The half-life of fenoxaprop in the control soil was 56.9 days which was much larger than that of fenoxaprop-ethyl (half-life 0.5 day). On the 35th day, large amounts of fenoxaprop still remain in the control soil with the concentration of 3.491 µg g^−1^.

### 3.2. Fate of fenoxaprop-ethyl in the biochar-amended soil

The effects of biochar on the fate of fenoxaprop-ethyl in soil are presented in figure 2. Fenoxaprop-ethyl also degraded fast in the biochar-amended soil. The degradation followed first-order kinetic equation *C_t_ =* 0.982 + 5.177 × 10^−0.758*t*^ with *R*^2^ of 0.968 (table 2). The half-life of fenoxaprop-ethyl in the biochar-amended soil was 1.2 days, which was longer than in the control soil (half-life 0.5 day). The possible reason was that biochar adsorbed fenoxaprop-ethyl and reduced the bioavailability and biodegradation of fenoxaprop-ethyl [14]. It took 2 and 17 days to degrade 90% in the control and biochar-amended soils, respectively. The application of biochar had significantly increased the residues of fenoxaprop-ethyl relative to the control soil. On the 35th day, the concentration was 0.742 µg g^−1^ in the biochar-amended soil and 10 times more than in the control soil (0.071 µg g^−1^). In short, biochar increased the residual risk of fenoxaprop-ethyl in soil.

Metabolite fenoxaprop first increased and then decreased in the biochar-amended soil (figure 3). Fenoxaprop reached the maximum at the 12th hour like in the control soil. However, the presence of biochar significantly reduced the residues of fenoxaprop in soil. The maximum residues were 3.865 and 5.787 µg g^−1^ in the biochar-amended and control soils, indicating a reduction over 33%. The degradation of fenoxaprop followed first-order kinetic equation *C_t_ =* 0.686 + 2.965 × 10^−0.620*t*^ with *R*^2^ of 0.953 (table 2). The half-life in the biochar-amended soil (1.5 days) was significantly shorter than in the control soil (56.9 days), probably because of the limited absorption of biochar to fenoxaprop and positive impacts of biochar on microorganisms. As far as we know, biochar did not provide additional sorption capacity for some herbicides in soil [25]. Fenoxaprop tended to be weakly bound to biochar in the study due to its good water solubility (61 000 µg ml^−1^) and low log *K*_ow_ (1.04) [26], which avoided the reduction of bioavailability to microorganisms. It has been widely reported that biochar could rapidly increase microbial abundance and alter community structure and function [27,28]. Distinct bacterial conditions may be observed in soils with and without biochar [29]. Biochar in the study probably benefited the degrading bacteria of fenoxaprop in soil and changed the biodegradation pathway. On the 35th day, fenoxaprop was significantly reduced in soil by the presence of biochar. Concentrations were 0.506 and 3.49 µg g^−1^ in the biochar-amended and control soils, respectively. The reduction was of over 85%. Therefore, biochar has the potential to reduce residue risk of fenoxaprop in soil.

### 3.3. Acute toxicity test

The toxicity of fenoxaprop-ethyl to soil organism earthworms (*E. fetida*) was evaluated by a 14-day soil test. All earthworms survived in the solvent control group soil at the end of test. In the experimental group soil, there was a dose–response relationship between concentration and mortality rate of earthworms. The mortality rates were 0%, 10%, 30%, 90% and 100% at concentrations of 62.5, 125, 250, 500 and 1000 µg g^−1^, respectively. Fenoxaprop-ethyl showed no effects on earthworm survival at concentration below 62.5 µg g^−1^. However, 1000 µg g^−1^ of fenoxaprop-ethyl caused 100% mortality. The calculated LC_50_ value was 322.9 µg g^−1^ (250.4–429.6 µg g^−1^), which indicated that fenoxaprop-ethyl was low toxic to earthworms.

No death was observed in the solvent control group biochar-amended soil at the end of the test. The results show that the toxicity of biochar to earthworms was limited, which was similar to that of the previous literature [22]. It has been reported that all the earthworms survived after 28-day incubation in soil amended with different kinds of biochar at a rate of 5% [22]. Earthworms would selectively ingest soil and avoid ingesting biochar [30]. In the experimental group biochar-amended soil, fenoxaprop-ethyl caused no death even at the maximum exposure concentration (1000 µg g^−1^), indicating that the LC_50_ value was above 1000 µg g^−1^. The LC_50_ values were 322.9 µg g^−1^ (250.4–429.6 µg g^−1^) in the experimental group soil and above 1000 µg g^−1^ in the experimental group biochar-amended soil. Based on LC_50_ values, biochar significantly reduced the toxicity of fenoxaprop-ethyl to earthworms (*p* < 0.01). The results may be due to the absorption of biochar. Biochar absorbed pesticides and led to prominent decrease in the bioavailability in soils to earthworms [31]. The reduced bioavailable fenoxaprop-ethyl and fenoxaprop were probably the main cause of the reduction in toxicity to earthworms. Therefore, biochar as soil amendment may not bring additional toxicity risk of fenoxaprop-ethyl to soil organism earthworms.

## 4. Conclusion

The fate of fenoxaprop-ethyl and metabolite fenoxaprop in the control and biochar-amended soils was investigated. The dissipation of fenoxaprop-ethyl was retarded by the biochar-amended soil. The half-lives of fenoxaprop-ethyl were 0.5 and 1.2 days in the control and biochar-amended soils, respectively. Less metabolite fenoxaprop was formed in the biochar-amended soil (maximum 3.865 µg g^−1^) compared to that in the control soil (maximum 5.787 µg g^−1^). The dissipation was much faster in the biochar-amended soil (half-life 1.5 days) than in the control soil (half-life 56.9 days). Biochar absorption and microorganisms condition were possible causes for the results. Earthworm was used as the representative of soil organism in the acute toxicity test. The amendment of biochar caused reduced toxic effects to earthworms. The LC_50_ values were 322.9 µg g^−1^ and above 1000 µg g^−1^ in control and biochar-amended soils, respectively. In short, biochar posed positive effects on residues and toxicity. The application of biochar to soil has good potential for remediation and may serve as a beneficial farming strategy for the soil environment.

## Data accessibility

Our data are deposited at Dryad: http://dx.doi.org/10.5061/dryad.09rd4 [32].
